# Dynamic Role of the G Protein in Stabilizing the Active State of the Adenosine A_2A_ Receptor

**DOI:** 10.1016/j.str.2018.12.007

**Published:** 2019-04-02

**Authors:** Sangbae Lee, Anita K. Nivedha, Christopher G. Tate, Nagarajan Vaidehi

**Affiliations:** 1 Department of Computational and Quantitative Medicine, Beckman Research Institute of the City of Hope, 1500 E. Duarte Road, Duarte, CA 91010, USA; 2 MRC Laboratory of Molecular Biology, Cambridge Biomedical Campus, Francis Crick Avenue, Cambridge CB2 0QH, UK

**Keywords:** G protein-coupled receptors (GPCRs), molecular dynamics simulations, adenosine receptor, ligand mobility, entropy, allosteric communication

## Abstract

Agonist binding in the extracellular region of the G protein-coupled adenosine A2A receptor increases its affinity to the G proteins in the intracellular region, and vice versa. The structural basis for this effect is not evident from the crystal structures of A_2A_R in various conformational states since it stems from the receptor dynamics. Using atomistic molecular dynamics simulations on four different conformational states of the adenosine A_2A_ receptor, we observed that the agonists show decreased ligand mobility, lower entropy of the extracellular loops in the active-intermediate state compared with the inactive state. In contrast, the entropy of the intracellular region increases to prime the receptor for coupling the G protein. Coupling of the G protein to A_2A_R shrinks the agonist binding site, making tighter receptor agonist contacts with an increase in the strength of allosteric communication compared with the active-intermediate state. These insights provide a strong basis for structure-based ligand design studies.

## Highlights

• GPCR conformation dynamics reveals the forward and backward allosteric mechanism
• Agonist binding increases the entropy in the intracellular region of the GPCR
• G protein binding shrinks the receptor-ligand contacts in the extracellular region
• Increased allostery between G protein and agonist in the GPCR-G protein complex

## Graphical Abstract

**Figure:**
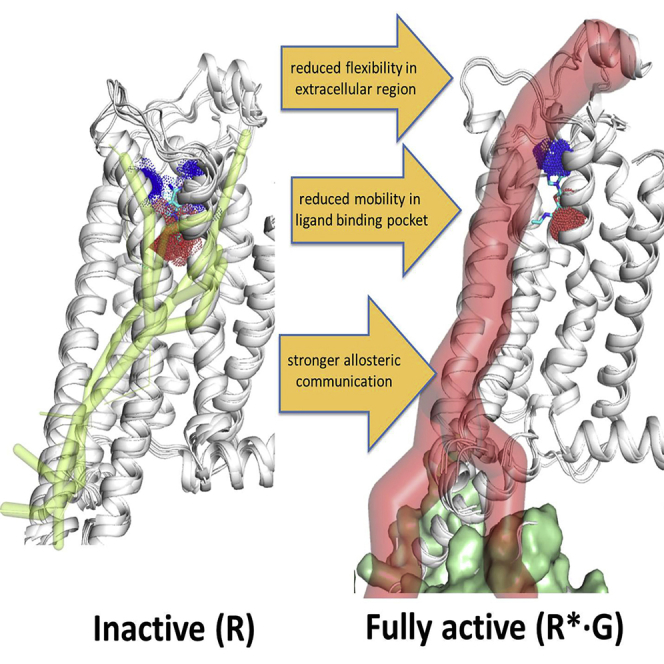

## Teaser

Lee et al. show that allosteric communication between the agonist binding site and G protein coupling site in GPCRs leads to enhanced agonist affinity. The agonist binding site shrinks dynamically when the G protein is bound to the receptor. Understanding GPCR allostery provides newer strategies to design selective ligands.

## Introduction

The adenosine A_2A_ receptor (A_2A_R) is a G protein-coupled receptor (GPCR) that is activated *in vivo* by the agonist adenosine (Fredholm et al., 2011). Subsequent coupling of the G protein, G_s,_ leads to the eventual increase in intracellular (IC) cyclic AMP through activation of adenylate cyclase and the modulation of downstream signaling pathways. A_2A_R is a validated drug target for the treatment of Parkinson's disease and cancer, which has resulted in its well-characterized pharmacology and a wide variety of synthetic agonists and antagonists (de Lera Ruiz et al., 2014). The structure of A_2A_R has been determined in the antagonist-bound inactive state (Cheng et al., 2017, Dore et al., 2011, Jaakola et al., 2008, Liu et al., 2012, Segala et al., 2016, Sun et al., 2017), the agonist-bound active-intermediate state (Lebon et al., 2011, Lebon et al., 2015, Xu et al., 2011), and in the fully active state bound to either mini-G_s_ (Carpenter et al., 2016) or to heterotrimeric G_s_ (Tate et al., 2018). The mechanism of activation conforms to the canonical paradigm (Rasmussen et al., 2011a) where agonist binding results in a slight contraction of the orthosteric binding pocket, rotamer changes of the hydrophobic gating residues Pro^5.50^-Ile^3.40^-Phe^6.44^ and opening of a cleft on the cytoplasmic face of the receptor primarily through an outward movement of transmembrane helix 6 (TM6). The C-terminal helix of G_s_, known as the α5 helix, binds in this cleft, resulting in nucleotide exchange and activation of the G protein (Carpenter et al., 2016, Rasmussen et al., 2011b).

The structure of A_2A_R in three defined conformations provides a series of snapshots during activation, but they do not provide information regarding receptor dynamics or the allosteric effect of G protein binding. The β_2_-adrenergic receptor (β_2_AR) is the most studied GPCR in terms of the dynamics of activation (Nygaard et al., 2013) and there are many similarities between A_2A_R and β_2_AR, but also some differences. The architecture of A_2A_R and β_2_AR is similar, they both couple to G_s_ and they both exist in an ensemble of conformations in the absence of an agonist (Manglik et al., 2015, Ye et al., 2016). In addition, coupling of G_s_ to the agonist-bound A_2A_R or β_2_AR increases the affinity of agonists at both receptors (Carpenter et al., 2016, Chung et al., 2011). In β_2_AR, it has been proposed that this increase in agonist affinity is a consequence of a closure of the entrance to the orthosteric binding pocket, resulting in a steric block to the exit of the agonist from the receptor (DeVree et al., 2016). The reason for the increase in agonist affinity in A_2A_R upon G protein coupling is unclear, because of the different energy landscapes of the respective receptors (Lebon et al., 2012). Crystal structures show that the agonist binding to β-AR stabilizes them in an inactive-like state (Sato et al., 2015). In contrast, agonist binding to A_2A_R results in conformational changes throughout the receptor into an active-intermediate state (Lebon et al., 2011) and only the outward bending of the cytoplasmic end of TM6 accompanies G protein binding (Carpenter et al., 2016). This is different compared with the receptor-wide changes observed in β_2_AR during the transition from the agonist-bound inactive state to the G protein-coupled state. Interestingly, the transition in A_2A_R from the active-intermediate to the fully active state does not involve any significant structural changes in the extracellular (EC) half of the receptor that defines the conformation of the ligand binding pocket (Carpenter et al., 2016). The similarities and differences in the various states of these two G_s_-coupled receptors depend on the dynamics and the energy landscape of these two receptors. Although the influence of the agonist in shifting the GPCR conformational ensemble has been studied (Manglik et al., 2015, Niesen et al., 2013, Nygaard et al., 2013), studies on the “reverse” influence of the ensemble of the GPCR-G protein complex on the agonist binding and the GPCR dynamics have been sparse. In addition, the role of allosteric effects in the GPCR when bound to both agonist and G protein is unclear. The crystal structures available in three different conformation states of A_2A_R offers a unique opportunity for studying the consequences of G protein coupling to a GPCR without the difficulties of decoupling the effects of the agonist from the effects of the G protein. We have therefore used atomistic molecular dynamics (MD) simulations on A_2A_R to understand the dynamic ensemble of conformations of the receptor in the inactive state, active-intermediate state, and the G protein-coupled fully active state to study the effects of the agonist and G protein on the receptor. Our results show that agonist binding to A_2A_R decreases the ligand mobility and entropy of the EC regions in the agonist-bound active-intermediate state compared with the agonist-bound inactive state. Importantly, the entropy of the IC regions increases upon agonist binding in the active-intermediate state compared with the inactive state, probably priming the receptor to bind to the G protein. Stabilization of the G protein-bound fully active conformation of A_2A_R shows increase in allosteric communication between the EC regions and the G protein-coupling IC regions. This reverse allosteric effect from the G protein to the ligand binding site explains the observed increase in agonist binding affinity to the G protein-coupled GPCR (DeVree et al., 2016, Carpenter et al., 2016).

## Results

Atomistic MD simulations were performed on A_2A_R bound to the agonists adenosine (ADO) or 5-N-ethylcarboxamidoadenosine (NECA), each in four different conformational states: (1) the inactive state of the receptor, R; (2) the active-intermediate state, R′; (3) the mini-G_s_ bound fully active state, R^∗^·G; and (4) a metastable state, R^∗^·G^−^, formed *in silico* by removal of mini-G_s_ from R^∗^·G. We also performed MD simulations on the inverse agonist ZM241385 bound inactive state and active-intermediate state of the receptor. To study the effect of Na^+^ ions on receptor dynamics, we performed MD simulations with Na^+^ ion in the ZM241385-bound inactive state, NECA-bound inactive, active-intermediate, and fully active states as detailed in Table S1 of the Supplemental Information. The initial structures for the simulations on R′ and R^∗^·G were from the crystal structures of A_2A_R bound to either NECA (PDB: 2YDV; Lebon et al., 2011) or NECA and mini-G_s_ (PDB: 5G53; Carpenter et al., 2016). The inactive state with NECA bound was generated from the crystal structure of A_2A_R bound to the inverse agonist ZM241385 (Dore et al., 2011) by replacement of the ligand with NECA followed by an equilibration protocol and MD production runs (see STAR Methods). The R^∗^·G^−^ state was generated by removing mini-G_s_ from the R^∗^·G state followed by equilibration and production runs. We used the crystal structure with Na^+^ ion bound for ZM241385-bound R state (PDB: 4EIY; Liu et al., 2012). The conformation for the R′ state of the wild-type A_2A_R generated above, was used to transfer the ZM241385 from the R state to the R′ state for further simulations. The list of systems simulated in this study, the notations used to represent different conformational states, and other details of these systems are given in Table S1. To analyze the conformation ensembles of A_2A_R in the different states, MD simulations totaling 1 μs (5 separate simulations of 200 ns each) were performed on the ligand-receptor or the ligand-receptor complex, with mini-G_s_, placed in explicit water and a lipid bilayer composed of palmitoyloleoylphosphatidylcholine. The results for A_2A_R bound to NECA are shown in the main text; similar results were obtained using adenosine and are all shown in the Supplemental Information (Figures S1–S5).

**Figure 1:**
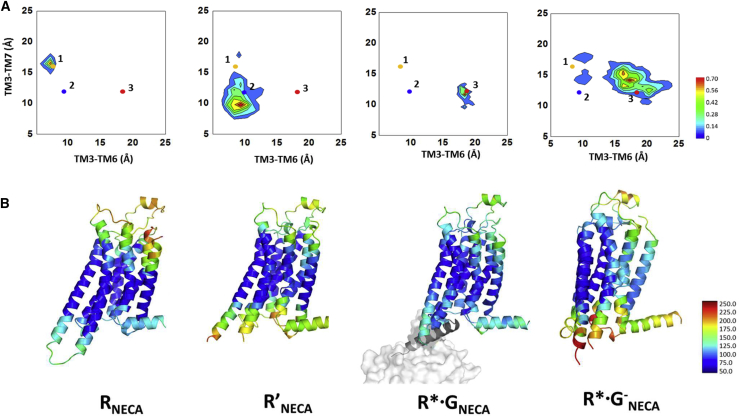
Conformational Sampling of A_2A_R Bound to NECA in Four Different States (A) Conformational ensembles from the MD simulations clustered by comparisons of the distances between TM3-TM6 and TM3-TM7. MD ensembles for A_2A_R bound to NECA in four different states were projected on to these two distances and contour maps plotted for the C_α_-C_α_ distances of R102^3.50^-E228^6.30^ and R102^3.50^-Y288^7.53^. The numbers 1, 2, and 3 in the figures correspond to the C_α_-C_α_ distances in the crystal structures of inactive (PDB: 3PWH, number 1), active-intermediate (PDB: 2YDV, number 2), and the mini-Gs-bound fully active state of A_2A_R (PDB: 5G53, number 3). (B) Representative structures extracted from the most populated cluster of A_2A_R bound to the agonist NECA in the inactive state (R_NECA_), the active-intermediate state (R′_NECA_), and the fully active G protein-bound state (R^∗^·G_NECA_). The R^∗^·G^−^_NECA_ state is a metastable state observed upon MD simulation of the receptor after removal of the G protein. The color scheme ranges from red to blue, with blue indicating low flexibility and red high flexibility. The flexibility is quantified by the B factor calculated from root-mean-square fluctuation in Å.

The conformations from the MD simulation trajectories were clustered by the C_α_-C_α_ distances between residues R102^3.50^ and E228^6.30^ on TM3 and TM6 and between R102^3.50^ and Y288^7.53^ on TM3 and TM7 (Figure 1), which are indicative of the receptor conformational changes upon activation (Tehan et al., 2014). It should be noted that these two distances are not the only measures of receptor activation. However, we use these two distances only to assess the breadth of the conformational sampling during MD simulations and not as a measure of receptor activation. An analysis of these distances for R_NECA_ and R^∗^·G_NECA_ showed well-defined values, whereas the equivalent distances in R′_NECA_ and R^∗^·G^−^_NECA_ exhibited a large spread of values. These data are consistent with increased flexibility and conformational heterogeneity of R′_NECA_ and R^∗^·G^−^_NECA_ compared with R_NECA_ and R^∗^·G_NECA_. The inverse agonist ZM241385-bound R and R′ states show narrow variations in the TM3-TM6 and TM3-TM7 distances (Figure S1C), consistent with the receptor being close to the starting R and R′ states respectively. Mapping the receptor flexibility calculated as root-mean-square fluctuation (RMSF) in Cartesian coordinates following superimposition of all frames onto the structures of A_2A_R showed that the most flexible region varies between the different conformational states. When NECA is bound to the inactive state (R_NECA_), the EC surface of A_2A_R is highly mobile, whereas the IC surface shows little variation in structure. In contrast, the R′ state is characterized by reduced mobility of the EC region and increased mobility of the IC region. G protein binding decreases the flexibility of the IC region, while the flexibility of the EC region remains the same. However, removal of the G protein to generate the R^∗^·G^−^_NECA_ state results in a highly flexible metastable state. The conformation ensemble for ADO bound to different states of A_2A_R shows a similar trend (Figures S1A and S1B).

### Binding Free Energy of Agonists Increases in the G Protein-Bound Fully Active State of A_2A_R

**Figure 2:**
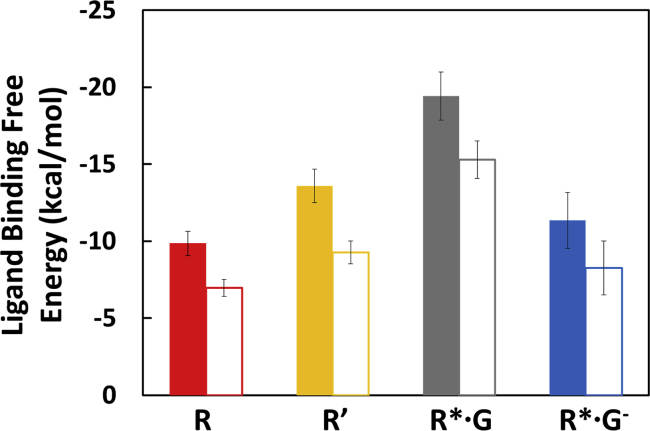
Free Energy of Agonist Binding to Conformational States of A_2A_R The binding free energies were calculated using the Bennett Acceptance Ratio free energy perturbation method (see the STAR Methods); NECA, colored bars; adenosine, open bars. The error bars are the SD.

Using the Bennett Acceptance Ratio method (see the STAR Methods) for calculating the difference in free energies between two systems, the free energy of binding was calculated for the agonists NECA and ADO to R, R′, R^∗^·G, and R^∗^·G^−^ conformations of A_2A_R. The binding free energy of both NECA and ADO is more favorable by 9.6 ± 0.8 and 8.3 ± 0.7 kcal/mol, respectively (Figure 2) in the fully active state, R^∗^·G, compared with the inactive state, R. The binding free energy of these two agonists is also more favorable in R^∗^·G compared with the R′ state, suggesting that the G protein coupling influences agonist affinity. This is corroborated by the decrease in affinity observed upon removal of the G protein in the R^∗^·G^−^ state (Table S2 of the Supplemental Information). It should be noted that the difference in calculated binding free energy is higher than the measured differences in binding affinity (Table S2 of the Supplemental Information). This discrepancy could be because the experimental binding affinity manifests from an equilibrium among multiple conformational states such as inactive and active-intermediate states, for example. Even the thermostabilized receptor will be in equilibrium between various conformational states in the presence of an agonist, including the inactive state and active-intermediate state. On the other hand, the MD simulations sample a smaller ensemble of states close to the starting conformational state. Therefore, the binding free energies calculated from the MD simulations reflect the ligand affinity to the specific conformational state of the receptor.

The average interaction energy of the ligand (agonist and inverse agonist) with the receptor, averaged over the MD trajectories, for each receptor conformational state is shown in Figure S2. Details of the calculation of the interaction energies are given in the STAR Methods. Both the agonists NECA and adenosine show highest interaction energy in the R^∗^·G state, while inverse agonist ZM241385 shows the highest interaction energy in the inactive R state. It is interesting to note that the agonist and inverse agonist do not show significant difference in their interaction energy in the active-intermediate R′ state.

**Figure 3:**
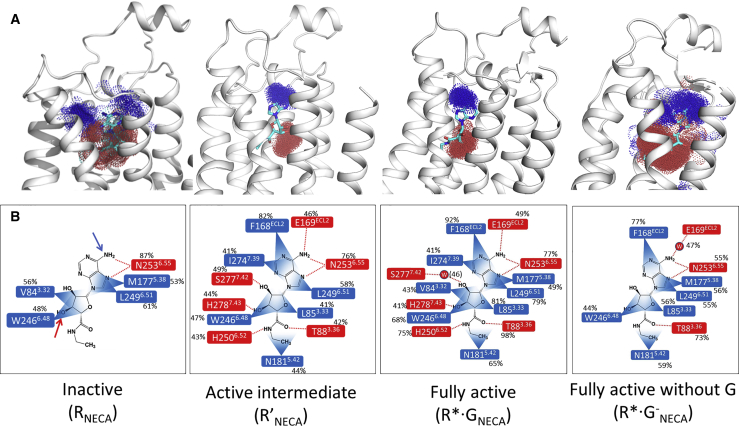
Mobility and Binding of NECA in Different Conformational States (A) Spatial distribution function of the agonist NECA calculated centering on the nitrogen atom from the primary amine group and the oxygen atom of the hydroxyl group in the sugar ring of NECA, blue and red arrows in (B) (see Figure S3 for data on adenosine). (B) The protein-ligand contacts for NECA binding in R_NECA_, R′_NECA_, R^∗^·G_NECA_, and R^∗^·G^−^_NECA_ states of A_2A_R. The protein-ligand contacts that are polar are marked in red and hydrophobic residue contacts are shown in blue. The percentage of snapshots within the MD simulations for each of these protein-ligand contacts is given (aggregated trajectory of 1 μs, 50,000 snapshots per calculation). N253 makes hydrogen bonds with two different N atoms on the adenine ring and the percentage shown is the sum of both.

The dynamics of agonists within the orthosteric binding site was assessed during the MD simulations to provide possible insights into why the affinity of NECA increases upon G protein coupling. The agonist movement and flexibility in the receptor was assessed by calculating the spatial distribution function for particular atoms in the agonists during the MD simulation (Figure 3A for NECA top panel, and Figure S3 for ADO; Table S3 of the Supplemental Information has a complete list of all the ligand-receptor contacts in all the conformational states and their relative populations) and also the RMSF from the average structure calculated from the MD simulations (Figure S4A). By both criteria, NECA shows high levels of movement within the R_NECA_ and R^∗^·G^−^_NECA_ states. In contrast, in the R′_NECA_ and R^∗^·G_NECA_ states, there appears to be far less movement of the agonists, suggestive of the orthosteric binding pocket being more rigid forming tighter ligand-protein contacts. The average volume of the agonist binding site remains similar in the R′ and R^∗^·G and R^∗^·G^−^ states, but there is a significant decrease in the volume upon transition from R to R′ (Figure S4C). Thus there is not a simple relationship between the volume of the orthosteric binding site and the degree of ligand movement. Similar trends in ADO flexibility was observed in different conformational states of A_2A_R (compare Figures 3 with S3).

The number of residues making sustained contacts with the agonist (present in greater than 40% of the MD snapshots) was significantly less in the R and R^∗^·G^−^ states compared with R′ and R^∗^·G (Figure 3B for NECA and Figure S3B for ADO), which might be expected given the different levels of ligand motion. Most of the A_2A_R-agonist contacts in the R′ and R^∗^·G state are preserved, although there are slight differences in contacts with V84^3.32^, M177^5.38^, and S277^7.42^, which show a low frequency of interaction below 40% of the MD snapshots in at least one of the structures.

### Entropy of the Extracellular and Intracellular Regions Varies between Different Conformational States of A_2A_R

**Figure 4:**
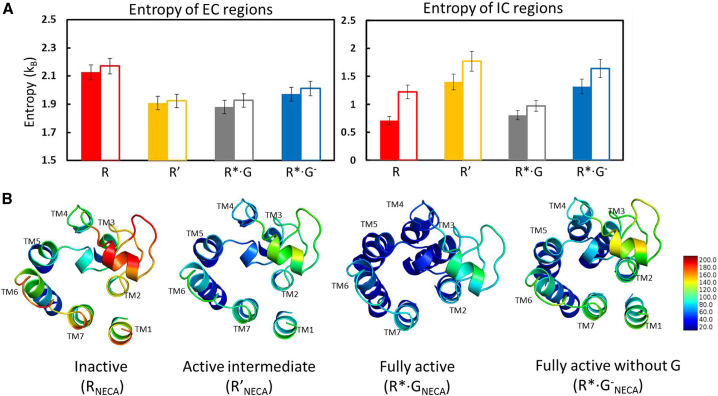
Entropic Effects of EC and IC Regions in Various Conformational States (A) Torsional entropy of the residues in the extracellular (EC) and intracellular (IC) regions of A_2A_R in various conformational states when bound to agonists NECA (solid colored bars) and ADO (white bars). The torsional entropy is shown in units of the Boltzmann constant k_B_. (B) The thermal B factor calculated from the root-mean-square fluctuations (RMSF) using the formula B factor = (8π^2^/3) (RMSF)^2^ of the C_α_ atoms in the EC regions are shown as a heatmap for the four conformational states of A_2A_R.

The torsional entropy was calculated for all the residues in the EC loops and the IC loops, including two turns of the adjacent TM helices (see the STAR Methods) for all the agonist-bound conformational states (Figure 4). The entropy of the EC region is highest in the R state, and this entropy decreases when the agonist stabilizes the R′ state. The entropy in the EC region in the R′, R^∗^·G and, R^∗^·G^−^ states are all similar. In contrast, the entropy of the IC regions is highest in the R′ and R^∗^·G^−^ states. As expected, G protein coupling in the R^∗^·G state significantly reduces the entropy of the IC region, especially in the IC loop 2 (ICL2) region (Figure S4D of the Supplemental Information), to a level similar to that observed in the R state. It should be noted that the reduced entropy could simply stem from the G protein binding to the IC regions of the receptor in the R^∗^·G state. The fluctuations in the RMSF of the C_α_ atoms of A_2A_R in the EC regions for the four conformational states of A_2A_R bound to NECA, reflect this trend in entropy (Figure 4B). The entropy of the individual EC and IC loops shown in Figure S4D of the Supplemental Information exhibits the same trend as the total entropy of the loops. The residues in the ICL3 loop are missing in the present simulations and we examined if this could contribute to the increased RMSF in ICL3 loop in the R′ state. We had previously published results on the dynamics of NECA-bound R′ state in which we modeled the entire ICL3 loop conformation (Lee et al., 2014). Comparison of the flexibility (RMSF) of the ICL3 loop residues from the present simulations of NECA-bound R′ state without the ICL3 loop, to the RMSF of the ICL3 loop residues from our previous work shows that the change in RMSF is minimal with or without the ICL3 loop residues (Figure S4B). This reaffirms our finding in Figure S4D that the flexibility of the IC loops is influenced by agonist binding in the R′ state compared with the R state.

### Allosteric Communication Pipeline Strength Varies in Each of the Conformational States of A_2A_R

**Figure 5:**
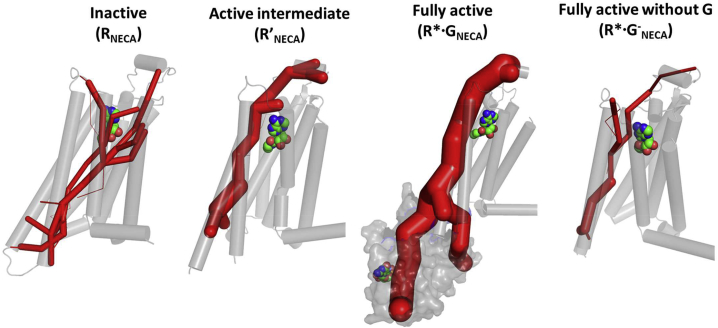
G-Protein Coupling Leads to Increase in the Strength of the Allosteric Coupling Allosteric communication pipelines from the EC region of the receptor to the G protein coupling region in the NECA-bound A_2A_R in various conformational states. The thickness of the pipelines shown is proportional to the strength of correlation in torsional angle motion of residues involved in this pipeline of communication.

Allosteric effects play an important role in communicating the effect of agonist binding to the G protein coupling region and vice versa. Therefore delineating the residues in the allosteric communication pipelines will provide vital information for designing drugs with selectivity. The strength of the allosteric communication pipelines was calculated in the agonist-bound R, R′, R^∗^·G, and R^∗^·G^−^ conformations using the program Allosteer (Bhattacharya et al., 2016, Bhattacharya and Vaidehi, 2014, Vaidehi and Bhattacharya, 2016) (see the STAR Methods). Binding of agonist or the G protein modifies the strength of the allosteric communication pipelines. The agonist-bound R′ state shows a strong allosteric coupling between the IC G protein coupling regions and the EC loop regions compared with the R state. However, G protein coupling to the receptor results in a dramatic increase in the strength of the allosteric coupling in R^∗^·G state (Figures 5 and S5 for ADO). The strength of allosteric communication reflects the level of correlated motion between residues in the EC and IC regions. Therefore, binding of both NECA and mini-G_s_ shows increased correlated motion in the receptor, thus stabilizing the fully active state.

### Effect of Na^+^ Ions on Various Agonist and Inverse Agonist-Bound Conformational States of A_2A_R

**Figure 6:**
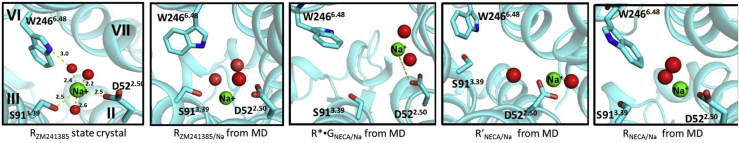
Effect of Na^+^ Ions in Diverse Conformational States Effect of Na^+^ ion in the MD simulations of the inverse agonist ZM241385-bound inactive state R (R_ZMA241385/Na_), agonist NECA-bound inactive state R (R_NECA/Na_), active-intermediate state R′ (R′_NECA/Na_), and G protein-bound fully active state R^∗^·G (R^∗^·G_NECA/Na_). The Na^+^ ion retains the hydrogen bonds with residues D52^2.50^ and S91^3.39^ and a water-mediated hydrogen bond with W246^6.48^ during the MD simulations of ZM241385-bound R state and as seen in the crystal structure of ZM241385-bound inactive state of A_2A_R (PDB: 4EIY). Agonist NECA binding in the R′ and R^∗^·G states disrupts the interaction of the Na^+^ with S91^3.39^ but retains the interaction with D52^2.50^. The waters present in the ZM241385-bound R state rearrange in the agonist-bound simulations.

An elegant structural study on the effect of Na^+^ ion on the inverse agonist-bound A_2A_R structure (Liu et al., 2012) showed that the Na^+^ ion attracts a cluster of well-ordered water molecules in its binding site near D52^2.50^ and S91^3.39^. This crystal structure also shows a water-mediated hydrogen bond between Na^+^ ion and W246^6.48^, as shown in Figure 6. We have compared the localized dynamics of the Na^+^ ion in the inverse agonist ZM241385-bound inactive state R (R_ZM241385/Na_), NECA-bound inactive state R (R_NECA/Na_), intermediate state R′ (R′_NECA/Na_), and fully active (R^∗^·G) state (R^∗^·G_NECA/Na_) (Figure 6). Figure 6 shows that during the MD simulations of ZM241385-bound R state, the Na^+^ ion makes sustained contacts with D52^2.50^ and S91^3.39^. However, in the NECA-bound inactive state R, the Na^+^ binding site becomes more flexible and the Na^+^ ion moves by 1.2 Å, possibly due to the change in the side chain rotamer angle of S91^3.39^ (Figures S6A and S6C of Supplemental Information). In contrast, the agonist NECA is less flexible and adopts a single conformation in the binding site in the R state (shown in Figures S6E and S6F of the Supplemental Information) when Na^+^ is present, compared with two conformations it adopts when Na^+^ is not present. Comparison of the ZM241385-bound R state to the NECA-bound R state shows no significant change in the side chain rotamer angle of D52^2.50^ (Figure S6A). D52^2.50^ shows a substantial shift in the rotamer angle in NECA-bound R compared with NECA-bound R′ state (Figures S6B and S6C). In all these simulations, the Na^+^ ion stays coordinated to the D52^2.50^ residue. The ordered waters observed in the Na^+^ binding site in the crystal structure of ZM241385 bound R state, become labile and do not stay close to the W246^6.48^ (Figure S6D) in all our MD simulations. In summary, the Na^+^ ion binding site becomes more flexible, loses the interaction with S91^3.39^ and the well-ordered waters get disrupted in the presence of the agonist in comparison with the inverse agonist. Figure S1D shows the flexibility of the IC region of NECA-bound A_2A_R in various conformation states when bound to Na^+^ ion. Comparing the results in Figures 1A and S1D, we observed that the Na^+^ ion located in the sodium binding site reduces the receptor flexibility in the IC region of the inactive and active-intermediate states, but not in the fully active state.

## Discussion

The structure of A_2A_R has been determined in three conformational states: an inverse agonist-bound inactive state, R (Dore et al., 2011), an agonist-bound active-intermediate state R′ (Lebon et al., 2011), and an agonist and G protein-bound fully active state R^∗^·G (Carpenter et al., 2016). These structures have been informative in elucidating the molecular basis for ligand recognition, and also opened up avenues to study the role of receptor dynamics and conformation ensembles of the agonist-GPCR-G protein complex and allosteric effects on the receptor emerging from the G protein binding. Here we have studied the dynamics of A_2A_R by MD simulations in various conformational states to give a deeper understanding of the conformation ensembles and the dynamical basis for high-affinity agonist binding.

Activation of A_2A_R is triggered by agonist binding and stabilization of the active-intermediate state, R′. However, the structural basis of why the agonist does not show favorable binding energy to the inactive receptor state (R) is not known and not feasible to measure experimentally, and so MD simulations were highly informative. Our MD simulations show that in A_2A_R, the agonist binding pocket in the R state has a larger volume than in the R′ and R^∗^·G. Thus, NECA bound to the R state shows high mobility and makes fewer sustained contacts to amino acid residues in the ligand binding pocket (contacts that are present in greater than 40% of the MD snapshots) than NECA bound to the R′ state. The EC surface is most mobile in the R state compared with other conformational states, while the IC surface is relatively less flexible due to the presence of the ionic lock between R102^3.50^ and E228^6.30^. The transition from the agonist-bound R state to the agonist-bound R′ state is accompanied by conformational changes throughout the whole receptor (Lebon et al., 2011). The volume of the orthosteric site decreases, the number of contacts between the receptor and ligand increases and there is a consequent decrease in ligand mobility and a decrease in entropy of the EC regions. In contrast, the entropy of the IC region increases and the IC end of TM6 shows increased mobility. This is consistent with the agonist priming the receptor for coupling the G protein in a cleft at the IC surface formed primarily by the outward movement of TM6. Finally, coupling of mini-G_s_ to the IC surface of A_2A_R increases the number of residues contacting the ligand for greater than 40% of the simulation time and, as expected, decreases the entropy of the IC region through forming contacts with mini-G_s_. However, there is no further change in the overall entropy of the EC regions or the volume of the orthosteric site upon G protein coupling.

The MD simulations of A_2A_R provide unprecedented insights into why there is an increase in the affinity of agonist upon G protein coupling. Pharmacologically, it is exceedingly difficult to measure the affinities of an agonist for single conformational states of a GPCR due to the dynamics of GPCRs and the inevitable change in the dynamics upon ligand binding. In addition, comparison of affinities between different laboratories may be difficult due to different experimental conditions for both receptor expression and ligand binding assays, both of which can have a profound effect on ligand affinities. However, taking these caveats into account, it is clear that there is an increase in agonist affinity upon G protein coupling of between 40- and 100-fold (Carpenter et al., 2016, Murphree et al., 2002). In addition, if the inactive state is stabilized through the binding of a nanobody to the IC surface of A_2A_R, agonist binding affinity decreases by nearly 400-fold (Hino et al., 2012). However, in these experiments, comparisons were made to the wild-type receptor and it is unclear what the affinity for the individual conformations are, because there will inevitably be a mixture of conformational states (Ye et al., 2016) that probably include R, R′, and perhaps R^∗^. We calculated the ligand binding free energies for agonists (adenosine and NECA) to the different conformational states and see an increase in affinity in the transition from R to R′ and a further increase from R′ to R^∗^·G.

The increase in agonist affinity from R to R′ may arise from a number of sources. First, there is an increase in the number of residues making contact to the agonist due to the contraction of the binding pocket and the associated conformational changes. Secondly, there is a decrease in entropy in the EC region that would be predicted to make the region more rigid. Decreased flexibility of this region would likely decrease the off-rate of the ligand and may therefore affect affinity. Finally, there is a slight change in structure of the EC loops that may partially occlude the orthosteric site, which could slow down the off-rate of a ligand, as suggested for the β_2_AR (DeVree et al., 2016).

**Figure 7:**
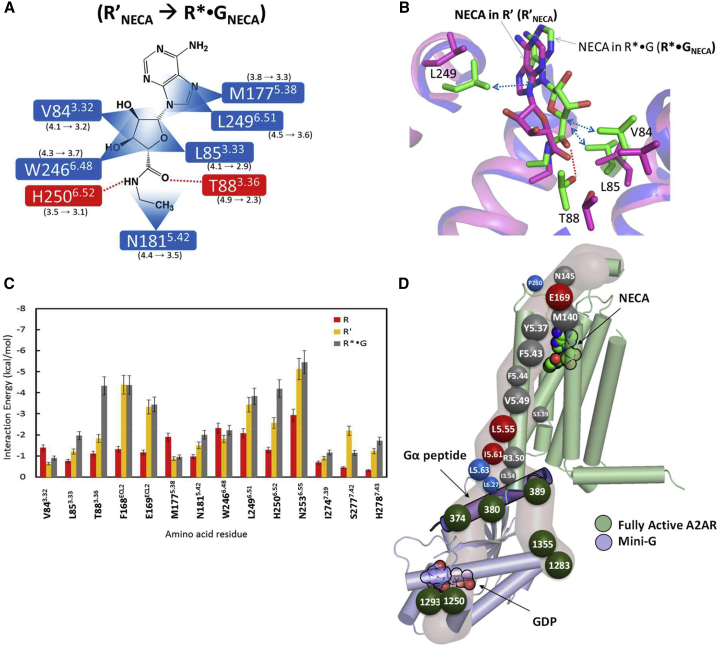
The Effect of G Protein Coupling in Increasing the Ligand Affinity Going from the Active-Intermediate R′ State to G Protein-Bound Fully Active (R∗·G) State. (A) The ligand-receptor contacts that showing over 20% increase in population between R′_NECA_ and R^∗^·G_NECA_ are shown. The numbers shown near each contact is the contraction in the average distance in each of these contacts going from R′_NECA_ to R^∗^·G_NECA_. (B) Representative structures of NECA binding site in R′_NECA_ (pink) and R^∗^·G_NECA_ (green) states with the residues that show significant contraction of ligand-residue distances in (A). (C) The non-bond interaction energy (kcal/mol) between agonist NECA and the residues in the ligand binding site A_2A_R in the inactive state (R, red), active-intermediate state (R′, orange) and fully active state (R^∗^·G, black). (D) The residues shown by their Ballesteros-Weinstein numbering scheme located in the allosteric communication pipeline from the EC region connecting the G protein-coupling residues via the ligand binding site. The size of the sphere is proportional to their strength contribution to the allosteric pipeline. Residues shown in gray spheres show reduced affinity for agonist when mutated to alanine, and those shown in blue spheres have an increased affinity for agonist, and the maroon sphere residues show less than 10% change in ligand binding upon mutation to alanine compared with the wild-type. The allosteric communication residues to the nucleotide (shown as outline in the figure) binding site in the G protein are shown in green spheres. The G protein numbering is taken from the PDB structure of A_2A_R with mini-G_s_ bound (PDB: 5G53). The A_2A_R receptor is shown in green and mini-G_s_ is in light blue.

The effect of G protein coupling to A_2A_R on the increase in agonist affinity in the transition from R′ to R^∗^·G is not clear from looking solely at the crystal structures of the respective conformations (Carpenter et al., 2016). The EC half of A_2A_R in the R′ to R^∗^·G conformational change does not undergo any significant structural change (0.3 Å RMSF for C_α_ atoms). Remarkably, the MD simulations show that not only the lifetime of the contacts between the receptor and agonist increase significantly (compare the percentage of the MD snapshots having the contacts shown in Figure 3B for R′_NECA_ and R^∗^·G_NECA_ conformations), but also the average distance between some of the contacts shortens by up to 2.6 Å (Figure 7A). It should be noted that this shift in average ligand-receptor distance for residues comes from the broad distribution of these ligand-residue distances in the R′_NECA_ state. This points to the observation that the ligand and the residues in the binding site are more flexible in the R′ state compared with the fully active R^∗^·G_NECA_ state (Figures 7B and S7). The residue N253^6.55^ shows closer contact with NECA in the R′ state compared with R^∗^·G state (Table S3 of the Supplemental Information). There is a small increase in favorable interaction energy between NECA and virtually every residue in the orthosteric binding pocket in the transition from R′ and R^∗^·G (Figure 7C), with the biggest increase observed for T88^3.36^. This can be described as a “velcro effect,” where the increase in agonist binding energy upon coupling of G_s_ binding is a consequence of multiple small increases in the free energy of interaction around the whole ligand. If the G protein is removed, then the mobility of the agonist increases due to a decrease in the number of ligand-receptor contacts thus decreasing the free energy of agonist binding.

The large effect of the G protein coupling to A_2A_R on the affinity of agonist binding is consistent with the increase in the strength of the allosteric pipeline from the nucleotide binding site in the G protein going through the G protein binding interface to the orthosteric ligand site in the receptor (Figure 7D). The allosteric communication is a pipeline of inter-connecting residues that show a high level of correlated movement (Bhattacharya et al., 2016, Bhattacharya and Vaidehi, 2014, Vaidehi and Bhattacharya, 2016). The allosteric hub residues shown in gray spheres in Figure 7D showed ≥10% reduction in NECA affinity when mutated to alanine. Residues shown in blue spheres have ≥10% increased NECA affinity upon alanine mutation, and the maroon sphere residues show less than 10% change in NECA binding upon mutation to alanine compared with the wild-type. The experimental results are from Dr. Tate's laboratory data tested for identifying thermostable mutations for A_2A_R in the agonist-bound R′ state. Most of these residues are allosteric to both ligand binding and G protein-coupling sites and possibly are involved in modulating the receptor function. Table S4 lists all the allosteric hub residues in the R, R′, and R^∗^·G states of the receptor. It should be noted that the mutations that reduce or increase the agonist binding affinity identified by Tate and coworkers, are not confined to the allosteric pipelines to the G protein coupling interface. This is understandable since there are other residues that could be involved in β-arrestin-coupling allosteric pipelines or other function(s) of the receptor. It should be noted that given sufficient simulation time the R^∗^·G^−^ state should transition and converge to R′ state. However, our goal here was to study the local ensembles of conformations of agonist-bound A_2A_R in different conformational states of the receptor.

The computational studies here have highlighted how the dynamics of the receptor in different conformational states is important in considering the causes of pharmacological phenomenon within the context of receptor structures. Given the high degree of structural conservation among GPCRs, it is highly likely that many of these observations will apply to other GPCRs. However, the magnitude of the effects may differ significantly given the potential differences between the energy landscapes of GPCRs and the free energy available upon agonist binding to elicit conformation changes. In particular, these computational studies show how a G protein allosterically stabilizes the orthosteric ligand binding site leading to an increase in agonist affinity.

## STAR★Methods

### Key Resources Table

**Table:** 

REAGENT or RESOURCE	SOURCE	IDENTIFIER
**Software and Algorithms**		

GROMACS5.1.0	(Hess et al., 2008)	http://www.gromacs.org/
PyMOL	(Schrodinger, 2010)	https://pymol.org/
VMD	(Humphrey et al., 1996)	http://www.ks.uiuc.edu/Research/vmd/

### Contact for Reagent and Resource Sharing

Further information and requests for resources and reagents should be directed to and will be fulfilled by the lead contact, Dr. Nagarajan Vaidehi, NVaidehi@coh.org.

### Method Details

#### Receptor Structure Preparation and Details of MD Simulations

All the MD simulations of wild type A_2A_R in four different states were performed using the GROMACS5.1.0 package (Hess et al., 2008) with the GROMOS force field (Oostenbrink et al., 2004). The initial coordinates of the inactive, active-intermediate, and fully active state, including the coordinates of the agonist NECA were taken from the PDB code 2YDV (Lebon et al., 2011), 3PWH (Dore et al., 2011), and 5G53 (Carpenter et al., 2016), respectively. The Inverse agonist ZM241385 bound A_2A_R-StaR2 thermostable mutant (PDB code 3PWH) in the inactive state and agonist NECA bound A_2A_R-GL31 mutant (PDB code 2YDV) contain eight and five mutations respectively (3PWH: A54L^2.52^, T88A^3.36^, R107A^3.55^, K122A^4.43^, L202A^5.63^, L235A^6.37^, V239A^6.41^, and S277A^7.42^ and 2YDV: L48A^2.46^, A54L^2.52^, T65A^2.63^, Q89A^3.37,^ and N154A^ECL2^). The thermostabilizing mutations were mutated back to the wild type residues using Maestro9.2. Residues within 5Å of the sites of mutation were minimized using MacroModel with position restraints on all backbone atoms and all residues outside 5Å from the site of mutation. For studying the effect of Inverse agonist ZM241385 and agonist NECA bound inactive state of the receptor on the sodium ion in the allosteric site we started the simulations from the crystal structure of ZM241385 bound inactive state of A_2A_R with PDB code, 4EIY, which contains a sodium ion at the allosteric site. Each of the prepared structure was minimized in energy using the steepest descent method in Gromacs. We retained all the crystal waters and added sodium and chloride ions to neutralize each system. For the simulations that examine the effect of the sodium ions we added 0.15M of NaCl. We used the SPC forcefield for the waters in the simulations (Berendsen et al., 1987). The list of each system simulated and the number of POPC, water molecules and sodium and chloride ions in each system are in given in Table S1.

##### Equilibration Procedure

Each solvated receptor system (listed in Table S1 of the Supplemental Information) was equilibrated using the following steps: (a) 200ps of MD using NVT ensemble followed by (b) 40 ns of MD simulations using NPT ensemble. During the NPT equilibration step the position restraints were gradually reduced from 5 to 0 kcal/mol/Å^2^ keeping distance ligand-binding site residue distance restraints constant at 5 kcal/mol/Å^2^. (c) In the final step of equilibration, we performed 10ns of unrestrained NPT simulations before starting the production runs. The SETTLE (Miyamoto and Kollman, 1992) and LINCS algorithm (Hess et al., 1997) were used for the bond and angle for water and all other bonds, using 2 fs time step.

##### Production Runs

We performed unrestrained MD simulations on thirteen systems (listed in Table S1), each 1 μs long using NPT ensemble at 310 K and 1atm pressure with 2fs time step. We performed five production runs, each 200ns long, with different starting velocities for each system.

All the representative structures shown in the figures were rendered using PyMOL (Schrodinger, 2010) and VMD (Humphrey et al., 1996). All the analysis reported here from the atomistic MD simulation trajectories were done using the 5x100 ns ensemble collected from the last 100ns of each of the 5 simulations for each system.

#### Calculation of RMSF and Heat Map

The root mean square fluctuation (RMSF) for every residue was calculated using *gmx rmsf* modules of GROMACS. To depict the extent of flexibility for all the systems, on the receptor structure as a heat map, we converted RMSF to thermal B-factor using B-factor = (8π^2^/3)(RMSF)^2^. The average structure calculated from the combined trajectories (5 trajectories) of the last 100ns for each system was used as the reference structure for the RMSF calculations.

#### Calculating the Ligand Binding Free Energy Using the Alchemical Free Energy Method - Bennett Acceptance Ratio (BAR)

We have calculated the binding free energy (ΔG) of adenosine and NECA in the four conformational states (R, R’, R^∗^·G, and R^∗^·G^-^) using the Bennett Acceptance Ratio (BAR) algorithm (Shirts and Pande, 2005) in the GROMACS package. BAR method combines the information normally used for forward and reverse free energy perturbations. This can be expressed as function of a coupling parameter, λ, which indicates the level of change that has taken place between two states (bound and unbound), the extent to which the Hamiltonian has been perturbed and the system has been transformed. Simulations conducted at different values of λ allow us to plot a ∂H/∂λ curve, from which ΔG is derived. Transformations from ligand-bound (λ = 0) to ligand-free (λ =1) in our study were performed in equidistant λ spacing of 0.05 (Δλ=0.05) from 0 to 1 for decoupling Coulombic and van der Waals interaction using 10ns of simulation for each window.

#### Calculating Mutual Information (MI) in Torsional Angle Distribution and Torsional Entropy

We have previously showed that the use of internal coordinates for conformational entropy reduces the fluctuations compared to using Cartesian coordinates (Killian et al., 2009). We have neglected the contributions from bond and angle degrees of freedom since they tend to be relatively small. First order conformational entropy was calculated using the Gibbs entropy (Killian et al., 2009) for each torsion angle. A correction for under sampling was applied as has been done previously described (Pandini et al., 2012, Steuer et al., 2002). For the equations used please refer to reference (Niesen et al., 2013).

#### Calculation of Allosteric Pipelines Using *Allosteer*

To calculate the allosteric communication pipelines, the MD simulation trajectories were used for each agonist-GPCR pair using *Allosteer* computational method (Bhattacharya et al., 2016, Bhattacharya and Vaidehi, 2014, Vaidehi and Bhattacharya, 2016) that we have developed previously. We first calculated the mutual information in the torsion angles for all residue pairs in the extracellular surface and the residues in the G-protein coupling surface using the residues listed in the table below (under the section titled Definition of the residues in the intracellular and extracellular regions). For this, the trajectories from the five 200ns of MD simulations (a total of 1μs of MD simulations) were used. Then, the shortest pathway with maximum mutual information starting from the extracellular going to the G-protein coupling region passing through the agonist binding site residues was calculated using graph theory algorithm in MATLAB. More details on this method is provided in reference (Bhattacharya and Vaidehi, 2014). The strength of an allosteric communication pipeline in Figure 5 is the number of overlapping allosteric communication pathways contained in the pipeline (Bhattacharya and Vaidehi, 2014, Vaidehi and Bhattacharya, 2016). The strength of the contribution by each residue located in the allosteric pipeline (shown in Figure 6C as maroon circles) is quantified by the number of allosteric communication pathways going through that residue.

#### Definition of the Residues in Intracellular and Extracellular Regions

We have calculated the torsional entropy of the residues (Figure 4) in the extracellular and intracellular regions of the receptor. The definition of the residues in the extracellular and intracellular regions is given in the table below. We included the residues in the top or bottom two turns of the TM helices in our definition of the intracellular and extracellular regions.

**Table:** 

	Loop Region 1	Loop Region 2	Loop Region 3
Extracellular	I64-L78	L137-V178	C254-Y271
Intracellular	A30-Y43	I104-A121	L208-H230

The residues used to calculate the allosteric communication pipelines shown in Figure 5, are listed in the table below.

**Table:** 

G-protein Interface (GPI) residues	R102, A105-G114, I200, A203, A204, Q207-M211, A231, S234, L235
Binding Site (BS) residues	I60, A81-V86, Q89, K153, S156, C159, G162, C166, D170, V171, V239, F242, A243, W246, S263, L267-Y271
Extracellular (EC) region residues	S6-I10, T68-F83, N145-Y176, T256-L272

The properties listed below were all calculated using the last 100ns of each 200ns MD run.

#### Calculation of Spatial Distribution Function (SDF)

The spatial distribution function used for characterizing the agonist movement in the binding site was calculated using the program tool *gmx spatial* in the GROMACS package. This represents the density of the nitrogen atom from the primary amine group indicated with a blue arrow in Figure 3, and O atom of the hydroxyl group in the sugar ring indicated with a red arrow in Figure 3. We have done the same atoms for both NECA and adenosine over the entire MD trajectories.

#### Volume of the Ligand Binding Site

We have calculated the volume of the agonist binding site in four different conformational states of A_2A_R across all the MD trajectories. We first defined a 12×12×12 Å^3^ box centered at the centroid position of the agonist structure after equilibration. After filling the defined box with grid points at 1 Å resolution, we systematically deleted the grid points that overlapped with any atom, based on the van der Waals clashes. A spherical region positioned at the box center with 4 Å radius was defined as the core region. These steps were carried out automatically by program POVME (Offutt et al., 2016, Wagner et al., 2017).

#### Calculation of Ligand-Receptor Contact Distances

These are the definitions and tools we sued for calculating the ligand receptor residue contacts from the MD simulations shown in Figures 7A and S3. We first identified all the receptor residues that make hydrogen bond or van der Waals contact with the ligand in more than 40% of the MD snapshots. Such contacts are called sustained contacts. The hydrogen bond contacts were calculated between heteroatoms (N, O) with a distance cutoff of 3.5 Å and angle cutoff of 120° using the gromacs tool *gmx hbond*. The van der Waals contacts were calculated with a distance cutoff of 4Å between any two carbon atoms using VMD tool *ContactFreq*.*tcl*. We then calculated the average distance between the ligand and each residue that showed sustained contacts.

#### Calculation of Ligand-Receptor Interaction Energy

To assess the strength of interaction of the ligand with the receptor, we calculated the non-bond interaction energy of the ligand with the receptor as the sum of the Coulomb and van der Waals interaction energies, averaged over the last 100ns of the MD trajectories, using *gmx energy* in the GROMACS MD package.

## Supplemental Information

Document S1. Figures S1–S7 and Tables S1–S4

Document S2. Article plus Supplemental Information
