# Supplementary material for: Dynamic Role of the G Protein in Stabilizing the Active State of the Adenosine A2A Receptor
Source: Structure. 2019 Apr 2;27(4):703–712.e3. doi: 10.1016/j.str.2018.12.007 (PMC6531377; doi:10.1016/j.str.2018.12.007)
Supplement: Document S1. Figures S1–S7 and Tables S1–S4 [file mmc1.pdf]

Structure, Volume *27*

Supplemental Information

Dynamic Role of the G Protein in Stabilizing the Active State of the Adenosine A_{2A} Receptor

Sangbae Lee, Anita K. Nivedha, Christopher G. Tate, and Nagarajan Vaidehi

Dynamic role of the G-protein in stabilizing the active state of the adenosine A_{2A} receptor

Sangbae Lee,[1] Anita K. Nivedha,[1] Christopher G. Tate,[2] and Nagarajan Vaidehi[1,3,*]

[1]Department of Computational and Quantitative Medicine, Beckman Research Institute of the City of Hope, 1500 E. Duarte Road, Duarte, California 91010, USA

[2]MRC Laboratory of Molecular Biology, Cambridge Biomedical Campus, Francis Crick Avenue, Cambridge CB2 0QH, UK

[3]Lead Contact

*Correspondence: NVaidehi@coh.org

Supplementary Information

Table S1. Related to STAR methods section – Receptor Structure Preparation and Details of MD simulations. Details of the systems for which simulations were performed in thus study. The simulations time given in the last column comes from 5 production MD simulation runs, each 200ns long, performed for each system.

System/State	Notations	PDB	Ligand	waters	POPC	ions	Time
Using agonist NECA							
Fully Active State with mini-G	$R^{*}\bullet G_{NECA}$	5G53	NECA	35,395	256	$12Cl^-$	1μs
Fully Active State without mini-G	$R^{*}\bullet G^{-}_{NECA}$	5G53	NECA	10,822	128	$11Cl^-$	1μs
Active intermediate state	R'_{NECA}	2YDV	NECA	10,812	128	$9Cl^-$	1μs
Inactive state	R_{NECA}	3PWH	NECA	10,849	128	$10Cl^-$	1μs
Using agonist adenosine							
Fully Active State with mini-G	$R^{*}\bullet G_{ADO}$	5G53	[a]ADO	35,392	256	$12Cl^-$	1μs
Fully Active State without mini-G	$R^{*}\bullet G^{-}_{ADO}$	5G53	ADO	10,823	128	$11Cl^-$	1μs
Active intermediate state	R'_{ADO}	2YDO	ADO	10,894	128	$9Cl^-$	1μs
Inactive state	R_{ADO}	3PWH	ADO	10,897	128	$10Cl^-$	1μs
Using inverse agonist ZM241385							
Active intermediate state	$R'_{ZM241385}$	2YDV	ZM241385	10,780	128	$9Cl^-$	1μs
Inactive state	$R_{ZM241385/Na}$	4EIY	ZM241385	10,835	128	$1Na^+$, $11Cl^-$	1μs
Using Na^+							
Fully Active State with mini-G	$R^{*}\bullet G_{NECA/Na}$	5G53	NECA	35,727	256	$1Na^+$, $13Cl^-$	1μs
Active intermediate state	$R'_{NECA/Na}$	2YDV	NECA	10,784	128	$1Na^+$, $12Cl^-$	1μs
Inactive state	$R_{NECA/Na}$	4EIY	NECA	10,827	128	$1Na^+$, $11Cl^-$	1μs

[a]ADO: Adenosine

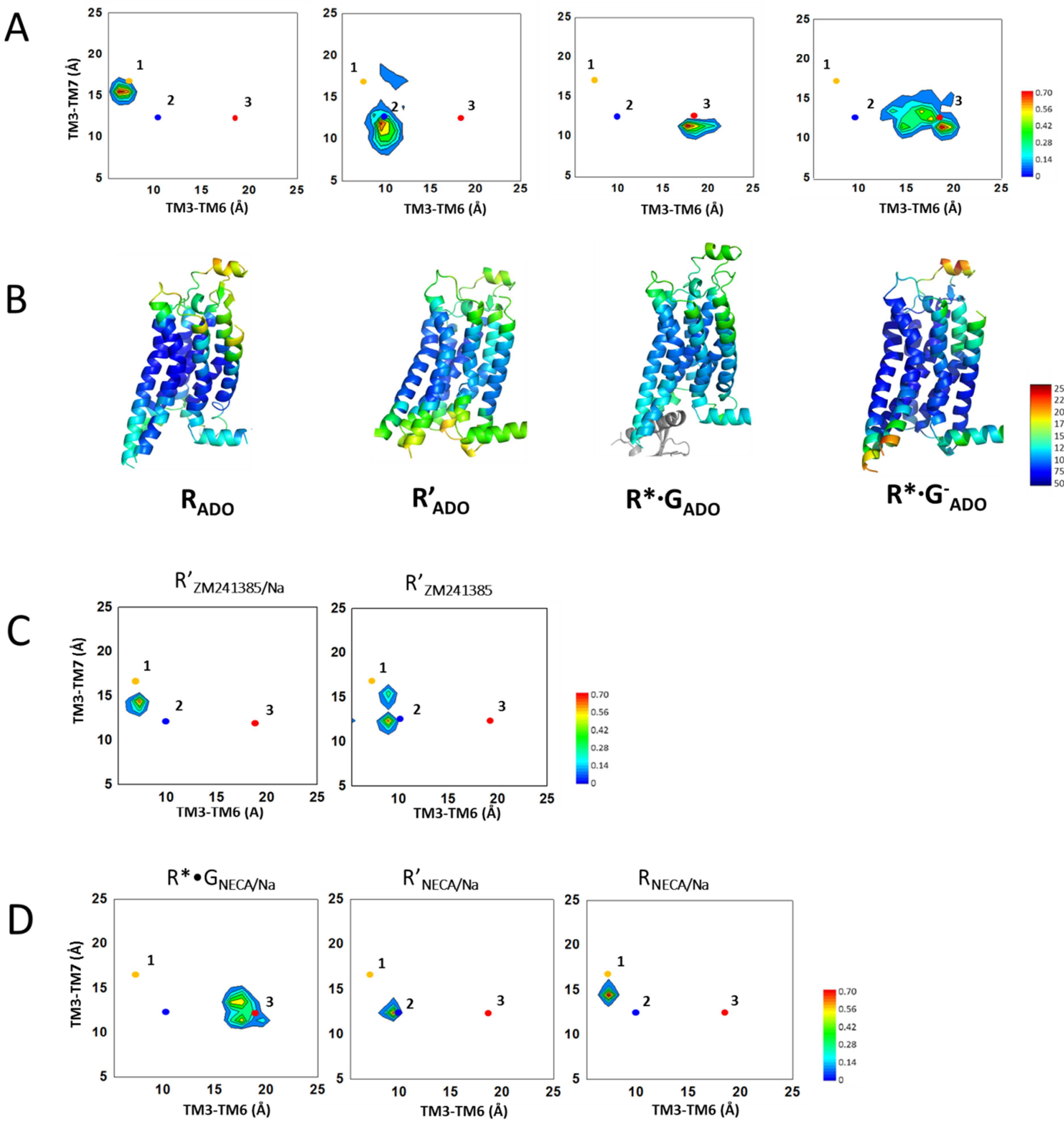

Figure S1. Related to Figure 1 and Figure 6. Structural homogeneity of the adenosine bound A_{2A} receptors in the four different states using (A) conformational ensembles by distance of C_{α}-C_{α} atoms between R3.50-E6.30 and R3.50-Y7.53, and (B) representative structures colored from low to higher flexibility or thermal B-factors calculated from RMSF (red to blue). (C) Conformational sampling of the inverse agonist ZM241385 bound inactive and active-intermediate states of A_{2A}R, and (D) conformational ensembles of agonist NECA bound fully active, active-intermediate, and inactive states in the presence of Na^{+} ions.

Table S2. Related to Figure 2. Experimentally measured binding affinities of NECA and Adenosine in $A_{2A}R$.

Ligand	pK_D (WT)	pK_D (StaR2)	pK_D (GL31)	Ref.
ADO	7.4	NA	7.5	2YDO
NECA	7.8	<5.5	7.9	2YDV
Ligand	pK_i (WT)	pK_i (mini-G)	pK_i (G_s+NB35)	Ref.
NECA	5.3	6.4	6.5	5G53

NA – not available

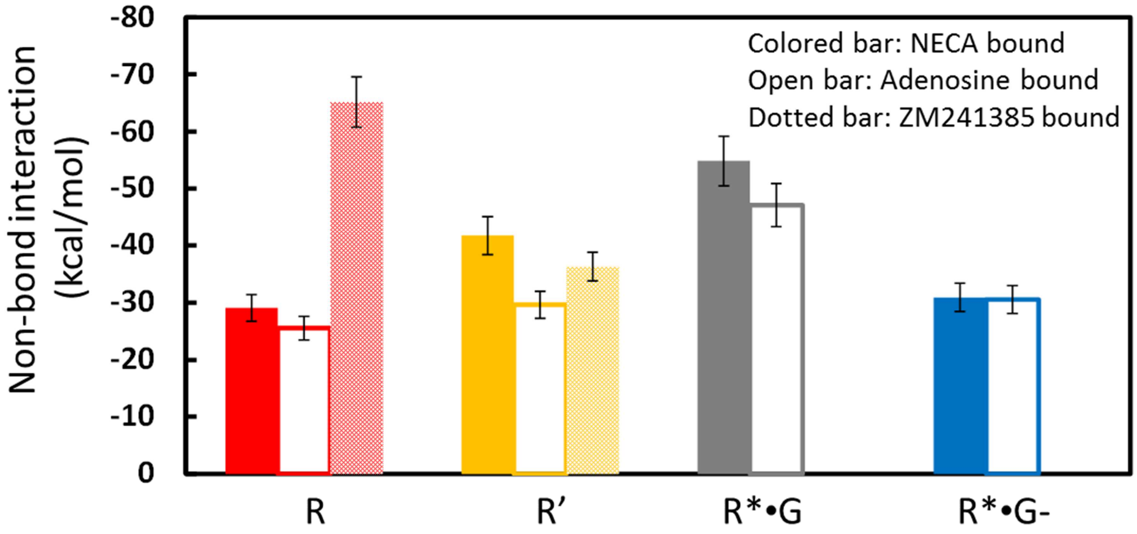

Figure S2. Related to Figure 2. Non-bond interaction energies of the full agonists NECA (sold colored bar), adenosine (open bar) and the inverse agonist ZM241385 (dotted bar) with residues in $A_{2A}R$ in different conformational states.

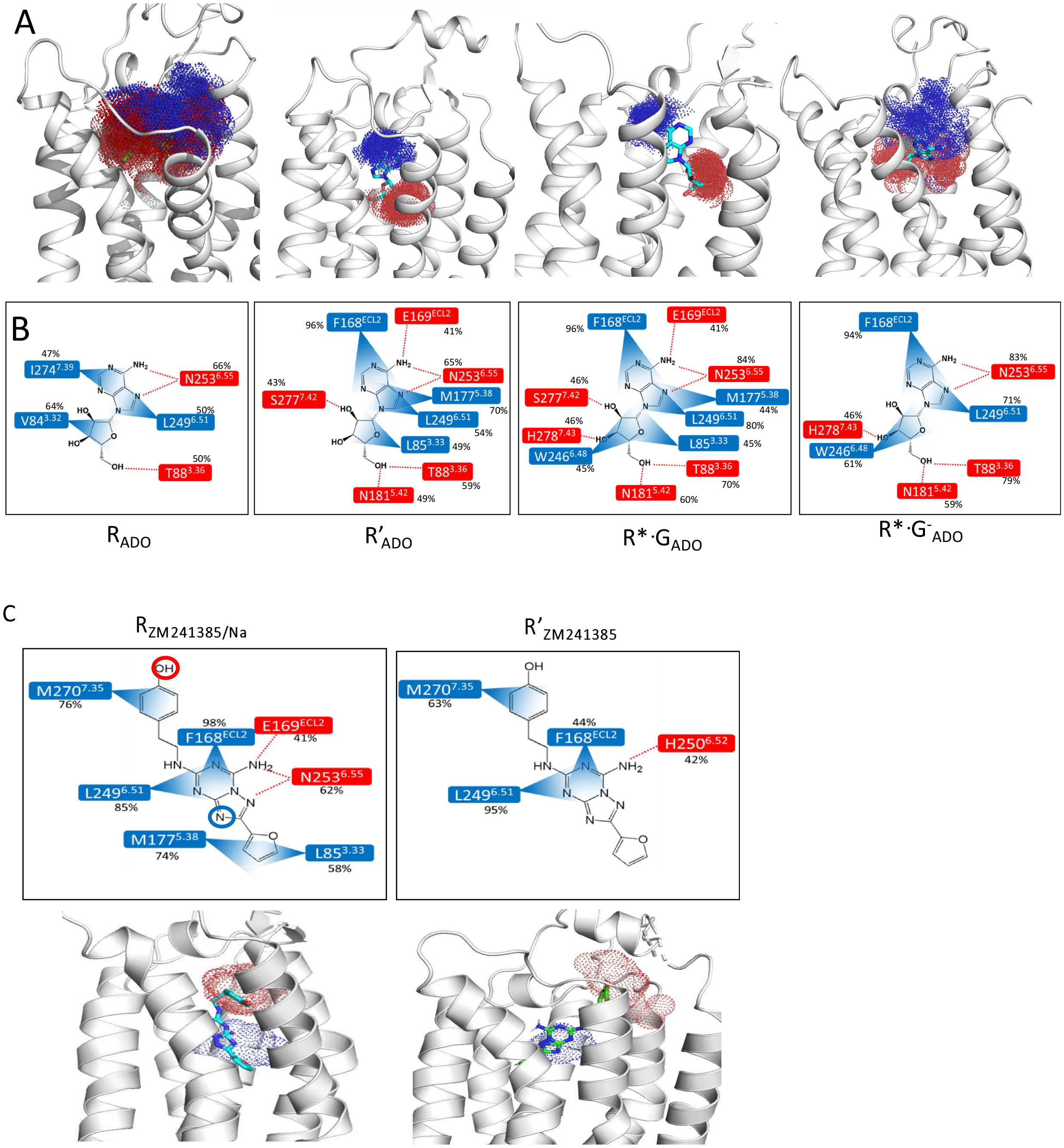

Figure S3. Related to Figure 3 and STAR methods section – Calculation of ligand-receptor contact distances. (A) Flexibility of the agonist Adenosine in the four different $A_{2A}R$ states. The structure of the ADO-receptor complex shown is the representative conformation from the most occupied conformational cluster. (B) The receptor-adenosine interactions for the R (inactive), R' (active-intermediate), R*•G (fully active with G-protein, and R*•G^{-} (fully active without G protein) states. (C) (Top) Interaction diagram of ZM241385 with receptor in $R_{ZM241385/Na}$ and $R'_{ZM241385}$ states. (Bottom) Flexibility of ZM241385 in the binding site.

Table S3. Related to Figure 3 and Figure 7. The percentage (%) of snapshots from MD that show the polar and nonpolar interactions between NECA-A_{2A}R and ADO-A_{2A}R in four different states, respectively. Blue and red highlights within receptor information indicate the van der Waals and hydrogen bond interactions, respectively. These contacts have been calculated based on crystal structure contacts. Other contacts formed during MD are **not shown here**.

NECA		R_{NECA}	R'_{NECA}	$R^{*}\bullet G_{NECA}$	$R^{*}\bullet G^{-}_{NECA}$
$V84^{3.32}$	C2’	56	23	43	29
$L85^{3.33}$	C4’	29	41	81	56
$T88^{3.36}$	O5’	3	42	98	73
$F168^{ECL2}$	C6	6	82	92	77
$E169^{ECL2}$	N6	9	46	49	47
$M177^{5.38}$	N7	53	31	49	56
$N181^{5.42}$	C52	27	44	65	59
$W246^{6.48}$	C3’	48	47	68	44
$L249^{6.51}$	C8	61	58	79	55
$H250^{6.52}$	N5’	1	43	75	30
$N253^{6.55}$	N1	41	69	77	55
$N253^{6.55}$	N6	46	76	47	41
$I274^{7.39}$	C2	18	41	41	34
$S277^{7.42}$	O2’	3	49	46	18
$H278^{7.43}$	O3’	1	44	41	12
ADO		R_{ADO}	R'_{ADO}	$R^{*}\cdot G_{ADO}$	$R^{*}\cdot G^{-}_{ADO}$
$V84^{3.32}$	C2’	64	33	32	29
$L85^{3.33}$	C4’	6	49	45	6
$T88^{3.36}$	O5’	50	59	70	78
$F168^{ECL2}$	C6	20	96	96	94
$E169^{ECL2}$	N6	14	41	41	14
$M177^{5.38}$	N7	10	70	44	10
$N181^{5.42}$	O5’	5	49	60	59
$W246^{6.48}$	C3’	1	27	45	61
$L249^{6.51}$	C8	50	54	80	71
$H250^{6.52}$	N5’	3	10	18	3
$N253^{6.55}$	N1	4	66	84	83
$N253^{6.55}$	N6	47	49	44	43
$I274^{7.39}$	C2	47	31	29	4
$S277^{7.42}$	O2’	5	43	46	5
$H278^{7.43}$	O3’	13	5	46	46

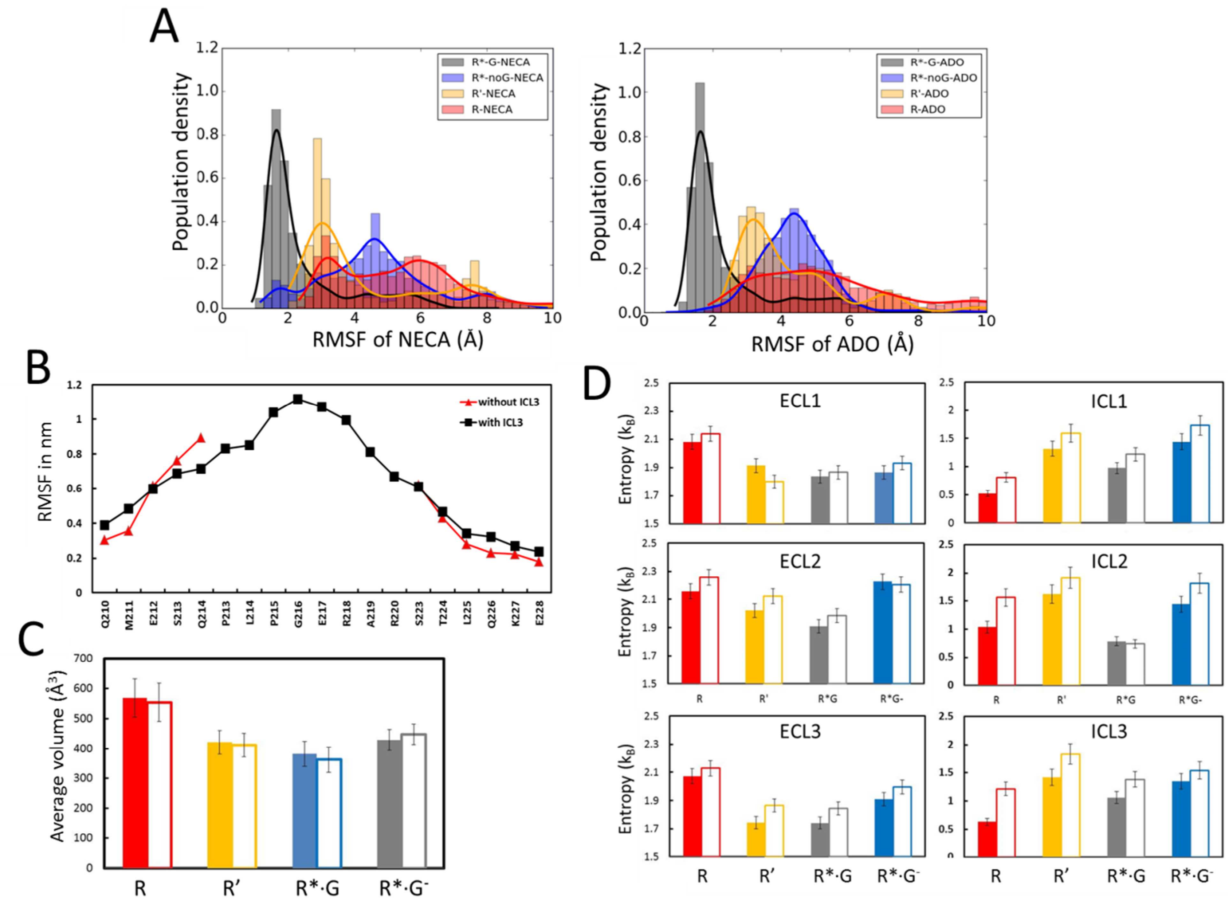

Figure S4. Related to Figure 3 and Figure 4. **A**. RMSF (Root mean square fluctuation) of the agonists NECA (left) and adenosine (right) from the average structure. **B**. RMSF of the residues in the ICL3 loop of NECA bound R' state with (black) and without (red – present simulations) all the ICL3 loop residues present. The values for RMSF for the black curve were calculated from our previously published MD simulations on NECA bound $A_{2A}R$ (Lee et al., 2014). **C**. The average volume of the agonist binding site in $A_{2A}R$ simulations calculated from the last 100ns of all the trajectories for each system; NECA, colored bars; adenosine, open bars. The error bars are the standard deviation of the aggregated trajectories for each system. **D.** Calculated torsional entropy for the residues in extracellular regions and intracellular loops. The solid bars are for NECA bound to different conformational states of $A_{2A}R$. Open bars are for adenosine bound states. Error bars are standard deviation calculated from the aggregated trajectories.

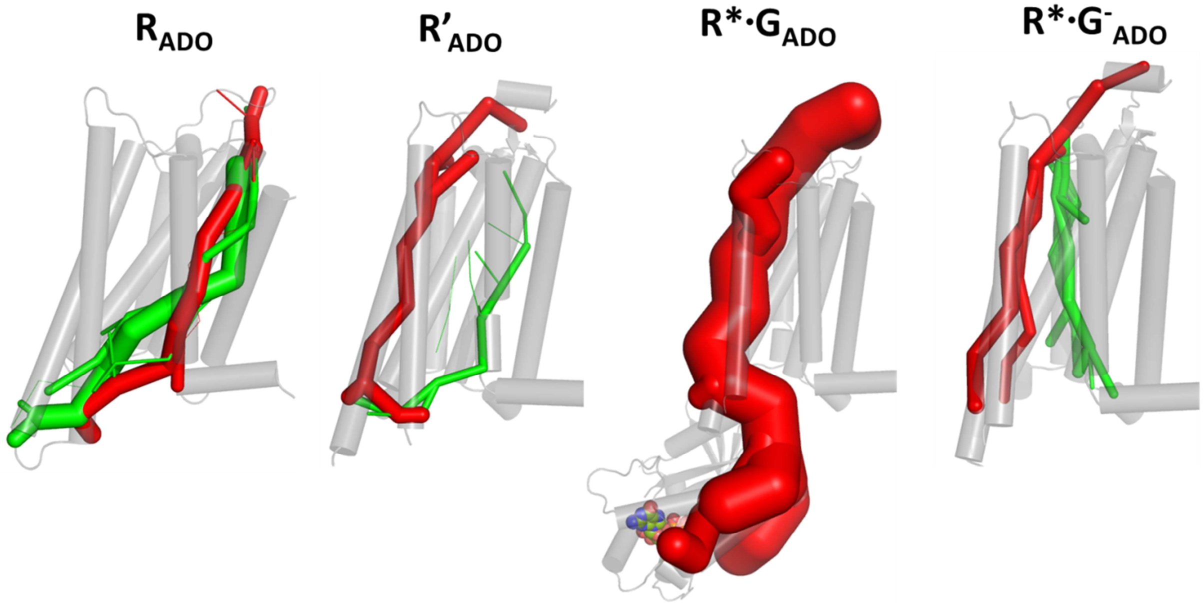

Figure S5. Related to Figure 5. Allosteric pipelines from the extracellular to G-protein binding regions in the adenosine bound $A_{2A}R$ in all four conformational states.

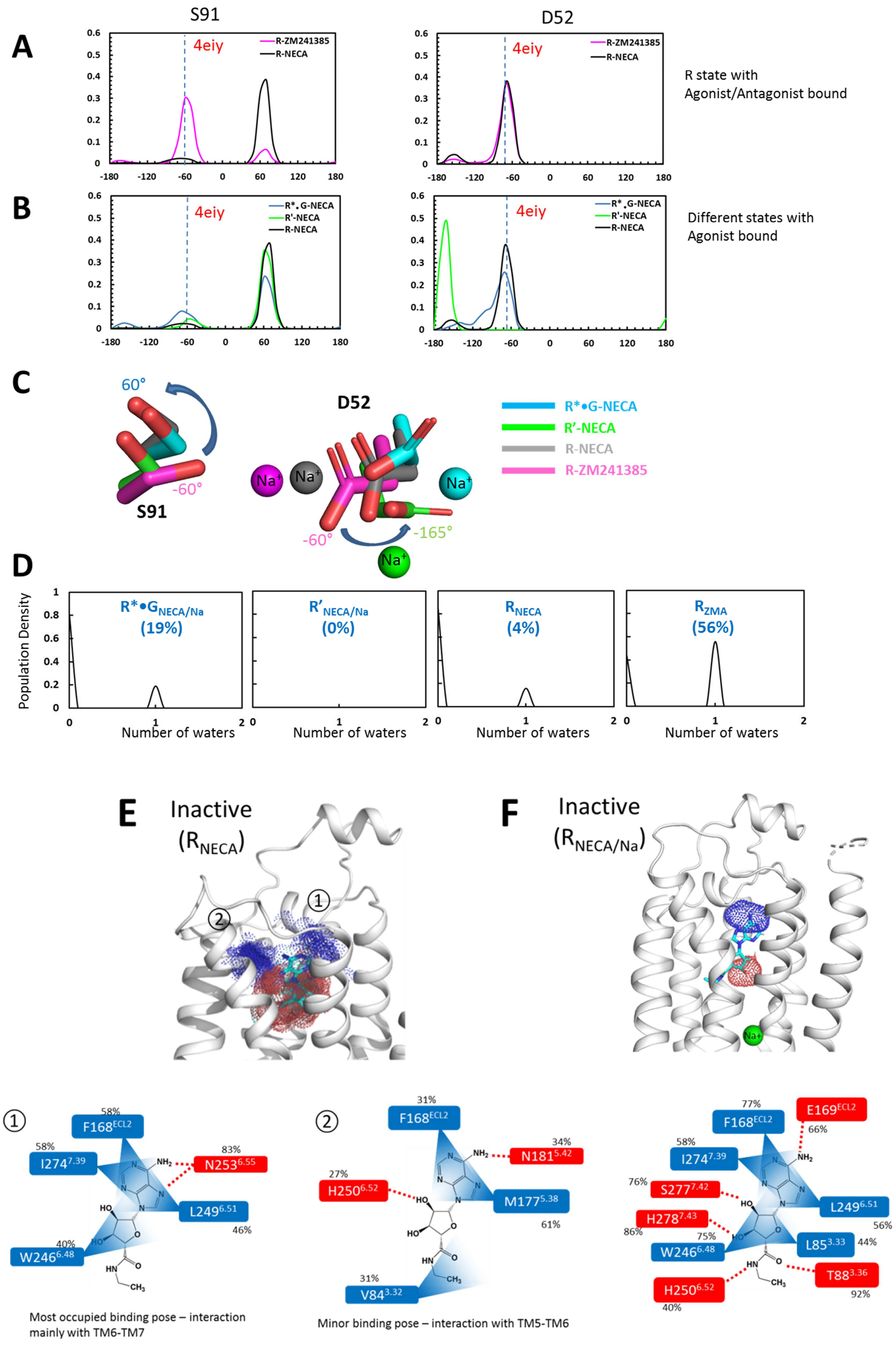

S91
D52
A
4eiy
R-ZM241385
R-NECA
R state with Agonist/Antagonist bound
B
R*.G-NECA
R'-NECA
Different states with Agonist bound
C
60°
-60°
S91
Na+
-165°
R*•G-NECA
R'-NECA
R-NECA
R-ZM241385
D
Population Density
Number of waters
(19%)
(0%)
(4%)
(56%)
E
Inactive
F
Na+
F168ECL2
I274 7.39
N253 6.55
L249 6.51
W246 6.48
N181 5.42
H250 6.52
M177 5.38
V84 3.32
E169ECL2
S277 7.42
H278 7.43
L85 3.33
T88 3.36
Most occupied binding pose – interaction mainly with TM6-TM7
Minor binding pose – interaction with TM5-TM6

Figure S6. Related to Figure 6. Effect of Na^+ ion on MD simulations of ZM241385 bound R, NECA bound to R, R' and R*•G states. For comparison we have shown our MD simulation results on the inverse agonist ZM241385 bound R state. **A**. The rotamer angle distribution of residues $D52^{2.50}$ and $S91^{3.39}$ from MD simulations of ZM241385 bound and NECA bound R state. **B**. The rotamer angle distribution of $D52^{2.50}$ and $S91^{3.39}$ from MD simulations of NECA bound R_{NECA}, R'_{NECA} and $R^*\bullet G_{NECA}$ states. **C.** The representative rotamers of $D52^{2.50}$ and $S91^{3.39}$ from different simulations. **D**. The population density of the MD snapshots that retain the water molecule that mediates the hydrogen bond with Trp246 shown in A. **E-F**. Effect of Na^+ ion on the NECA conformations in the binding site in the inactive R state of $A_{2A}R$. **E.** The MD simulations show two distinct conformations for the agonist NECA shown as ① and ② in Figure A. The most occupied conformation of NECA shows interactions with residues on TM6 and TM7 while the minor NECA conformation shows interactions with TM5 and TM6. **F**. NECA interaction in the binding site is stabilized by the presence of the Na^+ ion in the sodium binding site although the Na^+ ion itself is flexible when NECA is bound to the R state.

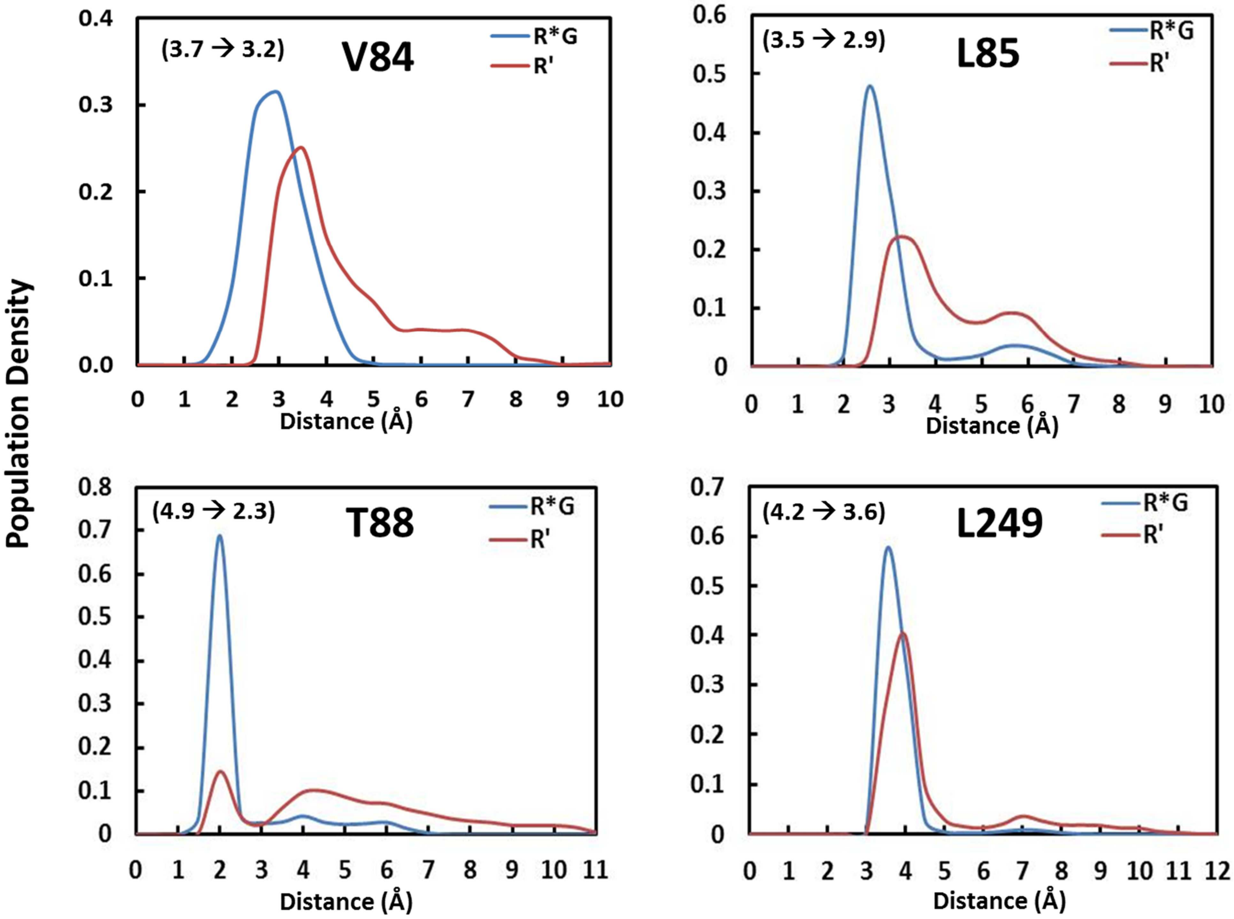

Figure S7. Related to Figure 7. Plot of population density of residue- NECA distances over the last 100ns of aggregated MD trajectories for NECA bound to R' and R*•G states. The residues V84, L85, T88 and L249 that show maximum contraction in ligand-residue distances are very flexible with broad distributions in the R' state compared to the R*•G state. This shows that the flexibility of the residues in the ligand binding site is higher in the active intermediate R' state compared to the fully active R*•G state.

Table S4. Related to Figure 7. List of predicted top scoring allosteric hub residues for NECA bound to fully active $R^{*}\bullet G_{NECA}$, active-intermediate state R'_{NECA} and inverse agonist ZM241385 bound inactive state $R_{ZM241385}$. The experimental effect on ligand binding was obtained from the thermostability measurements from Dr. Tate.

R*•G-NECA				R'-NECA				R-ZM241385			
BW #	Residue #	Mutation	NECA binding	BW #	Residue #	Mutation	NECA binding	BW #	Residue #	Mutation	ZM241385 binding
5.49	188	V188A	decrease	5.49	188	V188A	decrease	5.54	193	M193A	decrease
5.55	194	L194A	insignificant	5.55	194	L194A	insignificant	5.65	204	A204L	increase
5.43	182	F182A	insignificant	3.50	102	R102A	decrease	5.60	199	R199A	increase
5.30	169	E169A	m.d.	5.37	176	Y176A	m.d.	3.54	106	I106A	insignificant
5.37	176	Y176A	m.d.	5.43	182	F182A	insignificant	5.51	190	L190A	decrease
4.61	140	M140A	decrease	5.30	169	E169A	m.d.	ECL2	145	N145A	decrease
4.66	145	N145A	decrease	4.61	140	M140A	decrease	5.38	177	M177A	increase
3.50	102	R102A	decrease	ECL2	145	N145A	decrease	5.46	185	C185A	decrease
5.61	200	I200A	decrease	6.27	225	L225A	increase	3.48	100	I100A	decrease
5.63	202	L202A	increase	6.61	259	C259A	decrease	3.34	86	V086A	decrease
5.44	183	F183A	insignificant	6.55	253	N253A	increase	5.48	187	L187A	decrease
3.54	106	I106A	insignificant	5.61	200	I200A	decrease	6.32	240	G240A	increase
6.27	225	L225A	increase	3.54	106	I106A	insignificant	6.38	246	W246A	decrease
6.62	260	P260A	increase	5.64	203	A203L	increase	6.67	275	V275A	insignificant
3.39	91	S091A	decrease	4.70	149	P149A	increase	2.58	60	I060A	m.d.
2.59	61	P061L	m.d.	3.45	97	A097L	increase	2.64	66	I066A	m.d.
ECL2	146	C146A	m.d.	3.39	91	S091A	decrease	ECL2	150	K150A	insignificant
ECL2	148	Q148A	insignificant	2.65	263	S263A	increase	3.30	82	C082A	m.d.
6.35	233	K233A	decrease	2.60	62	F062A	increase	3.55	107	R107A	insignificant
3.33	85	L085A	m.d.	2.63	261	D261A	increase	5.49	188	V188A	decrease
m.d.: meaningless data due to expression issues; insignificant: insignificant change in agonist binding upon mutation;											
